# Hot Deformation Behaviour of Additively Manufactured 18Ni-300 Maraging Steel

**DOI:** 10.3390/ma16062412

**Published:** 2023-03-17

**Authors:** Błażej Tomiczek, Przemysław Snopiński, Wojciech Borek, Mariusz Król, Ana Romero Gutiérrez, Grzegorz Matula

**Affiliations:** 1 Scientific and Didactic Laboratory of Nanotechnology and Material Technologies, Faculty of Mechanical Engineering, Silesian University of Technology, 44-100 Gliwice, Poland; 2 Department of Engineering Materials and Biomaterials, Silesian University of Technology, 18A Konarskiego Street, 44-100 Gliwice, Poland; 3 Escuela de Ingeniería Industrial y Aeroespacial, Institute of Applied Aeronautical Industry Research, Universidad de Castilla-La Mancha (UCLM), 45071 Toledo, Spain

**Keywords:** additive manufacturing, hot deformation, 18Ni-300 maraging steel, microstructure

## Abstract

In this article, hot compression tests on the additively produced 18Ni-300 maraging steel 18Ni-300 were carried out on the Gleeble thermomechanical simulator in a wide temperature range (900–1200 °C) and at strain rates of 0.001 10 s^−1^. The samples were microstructurally analysed by light microscopy and scanning electron microscopy with electron backscatter diffraction (EBSD). This showed that dynamic recrystallization (DRX) was predominant in the samples tested at high strain rates and high deformation temperatures. In contrast, dynamic recovery (DRV) dominated at lower deformation temperatures and strain rates. Subsequently, the material constants were evaluated in a constitutive relationship using the experimental flow stress data. The results confirmed that the specimens are well hot workable and, compared with the literature data, have similar activation energy for hot working as the conventionally fabricated specimens. The findings presented in this research article can be used to develop novel hybrid postprocessing technologies that enable single-stage net shape forging/forming of AM maraging steel parts at reduced forming forces and with improved density and mechanical properties.

## 1. Introduction

Maraging steels (MS) are attractive alloys for engineers because of their superior mechanical properties, corrosion resistance, and excellent fracture toughness. They are usually classified into groups according to their 0.2% proof stress or yield strength ranging from 200 to 350 in ksi (1400 to 2400 MPa), e.g., M200, M250, M300, and M350, respectively. Maraging steels are produced by a two-stage vacuum melting process in which the chemical composition and impurity content are strictly controlled [1]. With a proper ageing treatment, ultra-high strength MS can be produced, suitable for the aerospace-, tool- and die-making industries [2].

Recently, maraging steels have become one of the most interesting materials in rapid prototyping due to their good printability and excellent response to LPBF [3,4,5]. High-quality, nearly fully dense parts have been successfully produced. Furthermore, studies have shown that the very high cooling rates and layer-by-layer deposition during the selective laser melting process enable the formation of a unique and very fine cellular structure in AM maraging steels [6]. As a result, they generally exhibit higher yield strength, tensile strength and hardness than conventionally manufactured parts [7].

Thermomechanical postprocessing of additively manufactured parts has recently been considered a promising way to exploit the advantages of both processes [8,9]. Higher performance levels have been shown to be achieved by combining AM with conventional postprocessing methods. For example, Arconic has introduced the AmpliforgeTM process [10]. This is a hybrid technology that combines the benefits of additive manufacturing and advanced forging processes to produce stronger, tougher parts with less time, cost, and material waste. On the basis of the positive results of this technology, more attention needs to be paid to leveraging the benefits of both processes to incorporate them into new process chains to achieve a more favourable cost structure and better-performing materials.

Compared to traditional manufacturing processes that require multiple steps (for example, forging or rolling), metal 3D printing is more straightforward. It enables the production of near-net shape components with complex geometries that require little additional machining effort [11,12]. However, it also has multiple disadvantages such as slow build rates and limited component size [13]. Hybrid postprocessing, which combines plastic forming and additive manufacturing, makes it possible to partially eliminate the disadvantages of both technologies and make the forming process more energy- and cost-efficient (for low-volume production) [14,15,16].

Considering that for some metals, the energy required to process the scrap is greater than that required for the 3D printing process [17], it is clear that it is more cost-effective to forge an additively manufactured part than a cast or wrought alloy. However, to apply the hybrid method, the deformation behaviour and mechanism at high temperatures must first be understood. Until now, the deformation behaviour and mechanism of conventionally produced M250 [18], M300 [19], and M350 [20] at high temperatures have been studied by compression tests. Despite extensive studies on the annealing process [21], selective laser melting parameters [22], and plastic deformation under quasi-static loading conditions of LPBF-MS [23], the intrinsic workability and microstructure control by high-temperature deformation of 18Ni-300 alloys produced by the LPBF technique have not been studied in detail.

Due to the uniqueness of the rapid prototyping processes and to exploit the full potential of the AM 18Ni-300 alloy, it is of great importance to evaluate the specific microstructural features resulting from post-fabrication thermomechanical processing [24]. This is conducted mainly by isothermal compression tests on solid cylindrical specimens in the laboratory to select the process parameters and establish the main material constants in the constitutive relations. Employing the experimental data derived from hot compression tests and performing numerical simulations on the worksamples, it is possible to estimate the strain, strain rate, and temperature under nonisothermal conditions while using constitutive equations to accurately predict the flow stress over a wide range of hot deformation conditions.

The current work is an attempt to characterise the hot deformation behaviour of the AM 18Ni300 alloy by studying the effects of the conditions of the hot compression process on the evolution of the microstructure. This work is intended to provide a template that can be followed during hot working of additively manufactured 18Ni-300 maraging steel. With this in mind, the present study was carried out with the following specific objectives:

- To analyse the stress–strain and work-hardening behaviour;
- To provide constitutive equations allowing prediction of flow stress behaviour of AM 18Ni-300 maraging steel;
- To determine the dominant microstructural restoration mechanism.

The originality of this work lies in the determination of the constitutive equation describing the hot working behaviour (hot flow stress–strain relationship) of additively manufactured 18Ni300 maraging steel, and in the analysis of the influence of hot working parameters on softening mechanisms such as DRV/DRX and other microstructural features, such as grain size and morphology, which can then be used to establish a correlation between hot working parameters and the resulting mechanical properties.

## 2. Materials and Methods

In this article, 18Ni-300 maraging steel powder produced by BÖHLER was deposited using Renishaw’s AM125 system to produce specimens. The process parameters given in Table 1 were used. The elemental composition of the spherical powder is given in Table 2. A meander scanning strategy was used with a rotation of 67° after the deposition of each layer to fabricate samples with a diameter of 6 mm and a height of 10 mm.

**Figure 1:**
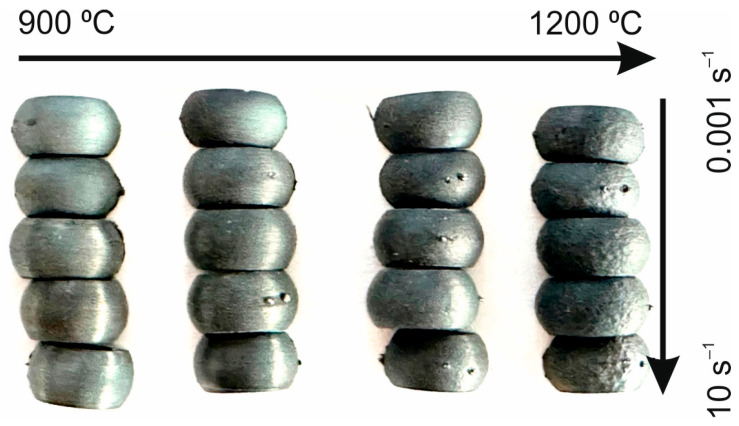
Comparison of the external surfaces of the hot compressed samples.

Isothermal compression tests were performed using a DSI (Dynamic System Inc.) Gleeble 3800 thermomechanical simulator on cylindrical specimens in the temperature range of 900 to 1200 °C with strain rates of 0.001, 0.01, 0.1, 1.0, and 10 s^−1^. Prior to deformation, the specimens were rapidly heated to the desired temperature and then held for 5 min to remove the temperature gradient. All specimens were compressed to a true strain of 0.7 and then to preserve the deformed microstructures, cooled to room temperature for 60 s with compressed air. Figure 1 shows the comparison of the external surfaces of the hot compressed samples.

The hot compressed specimens were cut along the compression axis and then prepared using a standard metallographic technique. Etching with a 10% Nital solution (10 mL of HNO_3_ + 90 mL C_2_H_5_OH) was used to reveal the details of the microstructure. Several sections of the specimens were polished and then viewed with an Axio Observer Z1 optical microscope (Carl Zeiss NTS GmbH, Oberkochen, Germany).

A field-emission Zeiss Supra 35 SEM microscope equipped with an EDAX EBSD system was used to analyse grain structure and orientation mapping. EBSD investigations were performed in a 200 × 200 μm^2^ area with an accelerating voltage of 20 kV, a step size of 0.5–1 μm, and a tilt angle of 72°. ATEX 4.02 software was used to generate IPF maps, estimate GND density, and postprocess the EBSD data.

## 3. Results

### 3.1. Microstructure before Deformation

**Figure 2:**
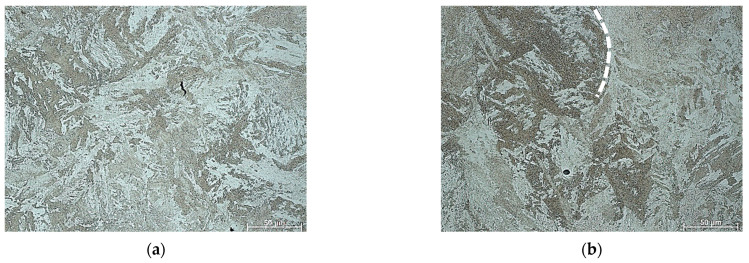
Microstructure of the 18Ni-300 maraging steel before deformation (**a**) cross-section (built) plane and (**b**) side plane.

Figure 2 shows the typical microstructures of the 18Ni-300 maraging steel in the as-built (SLM) condition. Chemical etching shows distinct process-related features, such as melt pool boundary (melt line) crossing a previously melted area (see white dashed line) and pores, which is typical for additively produced maraging steel. On the basis of the microstructural analysis, it can be determined that the starting microstructure is composed of columnar martensite lamellae and residual austenite phases.

**Figure 3:**
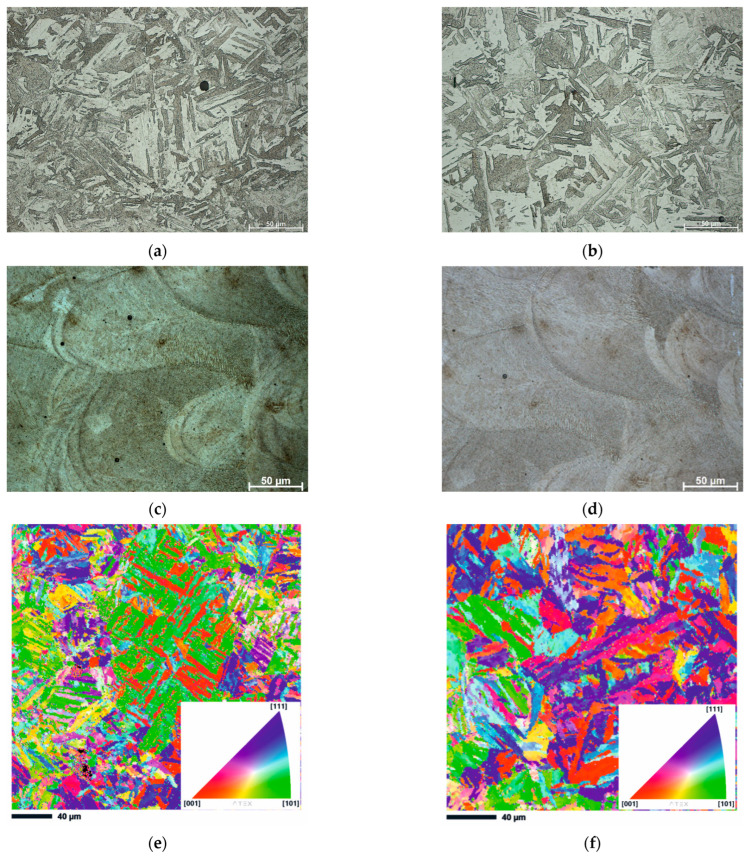

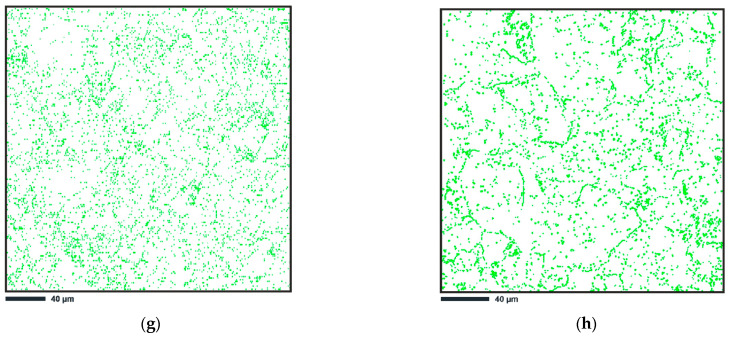
Microstructure of the 18Ni-300 maraging steel before deformation (**a**,**c**) after isothermal annealing at 900 °C, (**b**,**d**) at 1200 °C (**a**,**b**) etched in Nital and (**c**,**d**) saturated picric acid solution. Results of EBSD analysis and IPF-Z image of samples annealed at (**e**) 900 °C and (**f**) 1200 °C, IPF coloring triangles are shown in the lower right corners of figures, red [001], blue [111] and green [101], (**g**) grain boundary map (20–40°) of sample annealed at 900 °C, (**h**) grain boundary map (20–40°) of sample annealed at 1200 °C.

To investigate only the effect of heating on the deformation temperature on the microstructure of AM 18Ni300 steel, the samples were annealed for 5 min at 900 and 1200 °C degrees and then immediately quenched with water. Figure 3a,b show typical optical micrographs of AM 18Ni300 samples subjected to the abovementioned procedure. To determine the effect of this annealing on the microstructure, the samples were alternatively etched in two reagents. As can be seen, using Nital solution etching revealed a typical martensitic microstructure almost without visible melt pool boundaries. However, the second reagent, a picric acid solution, revealed the microstructure characteristic of materials produced by the SLM technique (Figure 3c,d). The boundaries of the laser scan traces can be seen where cellular/columnar subgrains are present. It is worth noting that even higher annealing temperatures do not allow significant microstructural homogenization, because with longer heat treatment time, the typical features such as “laser scan trace boundaries” should disappear. Despite the high temperature, the heating time was insufficient for complete homogenisation and recrystallization of the microstructure. Significantly, given that lath martensite transformed to austenite microstructure during heating to hot deformation temperature, identification of prior-austenite grains (PAGS) are much more important [5,25].

The issue of austenitization temperature has already been analysed by two of the co-authors of this paper. In investigated steel, the austenite formation begins around 622–642 °C and austenite transformation finishes at 818–825 °C, depending on the applied heating rate. More details about all transformations taking place in analysed material during the heating and cooling cycle can be found in work [26]. Revealing the prior-austenite grain boundaries can be based on EBSD analysis. It has been found that the angles, primarily in the range of 20°–40° in the case of martensitic structures, correspond to the boundaries of the prior-austenite grains. This range of misorientation angles was also used for our microstructure, Figure 3e,f. It should be highlighted that the misorientation angles in the mentioned range did not reveal a continuous envelope at the prior-austenite grain boundaries (Figure 3g,h). However, the obtained results enable manual PAGS reconstruction based on both 20°–40° misorientation angles. The EBSD analysis reveals a difference in PAGS in the range of 8–10 μm. Apparently, the short annealing time and the inhomogeneity of the structure, typical for additively produced samples, did not allow for the significant growth of the austenite grain.

### 3.2. Flow Stress Behaviour

**Figure 4:**
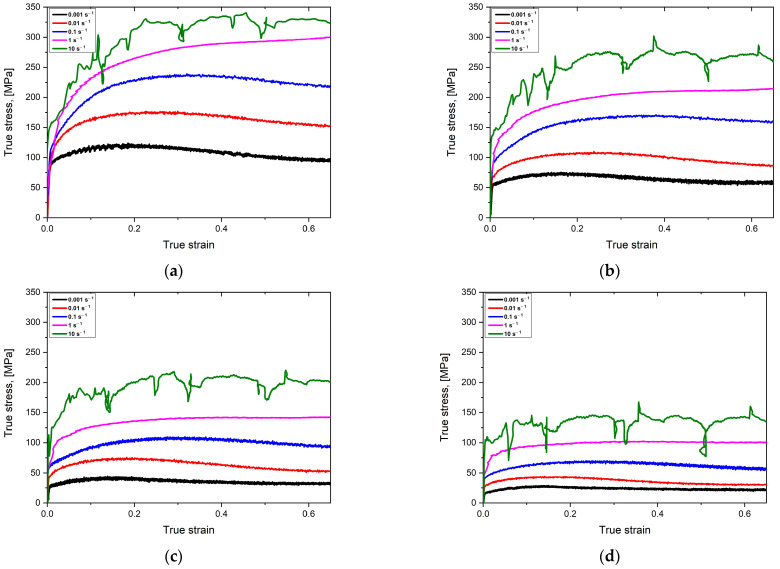
Typical stress–strain curves for the SLM M300 maraging steel obtained from uniaxial compression tests at various strain rates: (**a**) 900 °C, (**b**) 1000 °C, (**c**) 1100 °C, and (**d**) 1200 °C.

Figure 4 presents a series of typical stress–strain curves of AM 18Ni-300 maraging steel samples obtained under different deformation conditions during hot compression tests. It can be seen that the stress–strain curves at the strain rate of 10 s^−1^ show significant stress drops at all deformation temperatures, which may be related to the low sampling frequency of the Gleeble thermomechanical simulator during the experiments, as well as insufficient movement control capability for high strain rate tests. A significant problem may also be interference related to the constant temperature control test during deformation, and as is known, high-speed deformation causes an increase in energy and thus an increase in temperature so that the device tries to react in a very short time in order to stabilise the temperature at a certain level. In general, deformation at high speeds using the Gleeble simulator is burdened with various disturbances that we are not always able to eliminate directly on the device, and only reverse analysis allows us to partially eliminate some forms of disorders recorded during the tests. For this reason, the data from these curves were not included in the next analysis.

As expected, the decrease in the strain rate leads to a decrease in the flow stress. The main reason for this phenomenon is DRV (dynamic recovery) and DRX (dynamic recrystallization), which slow down the hardening at lower strain rates. Furthermore, the effect of the hot compression temperature on the flow stress is evident at any given strain rate. It can be clearly seen that the flow stress decreases significantly with increasing deformation temperature. This is due to the fact that the thermal activation and diffusion processes are more pronounced at high temperatures.

The next startling phenomenon is observed at a strain rate of 1 s^−1^. Here, the flow stress increases rapidly during the initial deformation phase, then a transition to a relative equilibrium state is observed, and finally, the flow stress increases slowly with the actual strain increase, Figure 4a,b. This behaviour indicates an unstable phenomenon due to competition between the dynamic softening mechanism and the strain hardening. Other stress–strain curves show a classical dynamic recrystallisation (DRX) with a single stress peak followed by a gradual downward trend toward equilibrium.

According to Figure 4c,d, deformation at 1100 and 1200 °C at a strain rate of 1 s^−1^ results in largely flat flow stress without significant strain hardening or softening, indicating that DRV is the dominant softening mechanism. The remaining stress–strain curves show classic DRX behaviour with a single stress peak followed by a gradual decrease in flow stress with increasing strain.

### 3.3. Constitutive Analysis

Constitutive equations are usually derived to determine the relationship between hot deformation parameters such as flow stress and strain rate. This relationship is usually expressed by the Arrhenius-type constitutive equation. The exponent-type Zener–Hollomon parameter (*Z*) is mostly employed to characterise the correlation between hot compression temperature and strain rate on the flow behaviour of most metals. This parameter is expressed as follows:

(1) $$Z=\dot{\varepsilon }\exp\left(\frac{Q}{RT}\right)$$

In the above equation, $\dot{\varepsilon }$ is the strain rate (s^−1^), *Q* is the DRX activation energy (kJ/mol^−1^), *R* is the universal gas constant (8.314 J/mol K), and *T* is the hot compression temperature (K).

Then the Arrhenius-type equation is used to show the influence of main deformation parameters on the flow stress behaviour. This equation is expressed as follows:

(2) $$\dot{\varepsilon }=\left\{\begin{array}{cc} A_{1}\sigma^{n_{1}} & \;\alpha \sigma <0.8\\ A_{2}\exp\left(\beta \sigma \right) & \;\alpha \sigma >1.2\\ A[\sinh{\left(\alpha \sigma \right)]}^{n}\exp\left(-\frac{Q}{RT}\right) & \;for\;all\;\sigma \end{array}\right.$$

**Figure 5:**
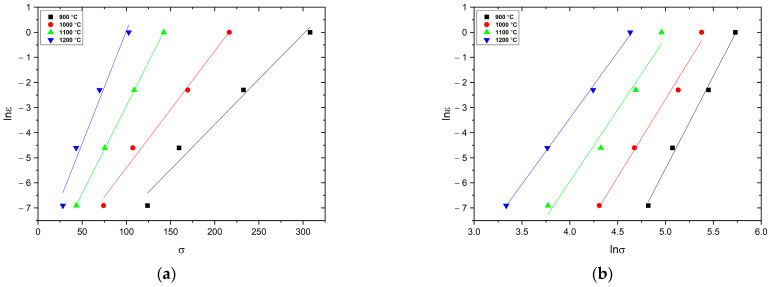
Relationship between ln(*ε*) and (**a**) *σ*, (**b**) ln*σ*.

Here, *A*, *n*, *n*_1_, *α*, and *β* are material constants that are independent of temperature and strain rate, and *σ* (Mpa) is the peak stress. Accordingly, the material constants *n*_1_ and *β* are derived from the slopes of $ln\left(\dot{\varepsilon }\right)-\sigma$ and $ln\left(\dot{\varepsilon }\right)-ln(\sigma )$, respectively, as shown in Figure 5a,b. Here, *n*_1_ and *β* are calculated as 6.13 and 0.06 Mpa^−1^, respectively. The adjustable scaling factor *α* can then be calculated using the following formula *α* = *β*/*n*_1_, which gives an *α*-value of 0.0098, Table 3.

**Figure 6:**
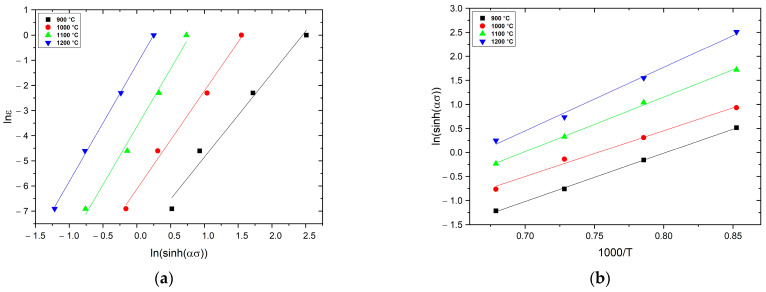
Relationship between (**a**) $ln\left(\dot{\varepsilon }\right)$ and lnsinh(*ασ*) and (**b**) lnsinh(*ασ*) and 1000/*T*.

The *n* parameter can be derived from Equation (3), by conducting a linear fitting of $ln\left(\dot{\varepsilon }\right)$ against ln(sinh(*ασ*)), Figure 6a.

(3) $$n={\left[\frac{\partial ln\left(\dot{\varepsilon }\right)}{\partial ln[\sinh\left(\alpha \sigma \right)]}\right]}_{T}$$

Then on the basis of Equation (2), at the constant of $\dot{\varepsilon }$, the activation energy (*Q*) can be expressed as follows:

(4) $$Q=Rn\frac{\partial ln[\sinh\left(\alpha \sigma \right)]}{\partial (1/T)}$$

In this equation, the $\partial ln(\sinh\left(\alpha \sigma \right))/\partial (1/T)$ value (S) is obtained by plotting the $ln(\sinh\left(\alpha \sigma \right))$ against (1/T) at different $\dot{\varepsilon }$ and conducting a linear fitting, Figure 6b. Substituting the calculated values of *n* and *α* into Equation (4), the value of *Q* can be derived.

Then the parameter *A* can be calculated on the basis of the following equation:

(5) $$\ln Z=\ln A+n\ln(\sinh\left(\alpha \sigma \right))$$

**Figure 7:**
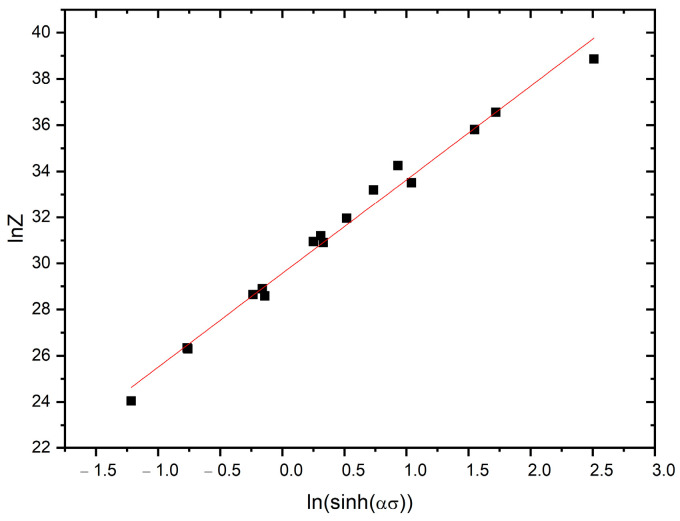
Relationship between ln *Z* and ln(sinh(*ασ*)) for the AM 18Ni-300 maraging steel (R2 value of 0.99).

The *A* value can be determined on the basis of the intercept of the linearly fitted line in the ln*Z* vs. ln(sinh(*ασ*)) plot, Figure 7.

The values of *A*, *n*, and *Q* are derived using the formulas above, and when they are substituted into Equation (4) they give the following constitutive relationship for the hot working of AM 18Ni-300 maraging steel:

(6) $$\dot{\varepsilon }=7.81\cdot 10^{12}[\sinh{\left(0.0098\sigma \right)]}^{4.13}\exp\left(-\frac{379\cdot 10^{3}}{RT}\right)$$

According to the calculations performed in this research article, the deformation activation energy of the additively fabricated maraging steel 18-Ni300 is 379 kJ mol^−1^. It is apparent that the calculated here activation energy value for AM 18Ni300 steel is similar to that of conventionally manufactured M300 grade (391 kJ/mol) [19], M350 grade (371 kJ/mol) [20] and is much lower than that of CF250 grade (458.8 kJ/mol) [27] in magnitude. Further, the activation energy value for AM 18Ni300 value is much larger than the values for self-diffusion in γ-iron (*Q* = 280 kJ mol^−1^), implying that dynamic recovery and dynamic recrystallization are the dominant mechanisms instead of diffusion during hot deformation [28]. It is also worth noting that the calculated *Q* value for the maraging steel AM 18Ni-300 is similar to that of the conventionally produced grade M300 (*Q* = 390 kJ mol^−1^) [19].

### 3.4. Microstructure after Deformation

**Figure 8:**
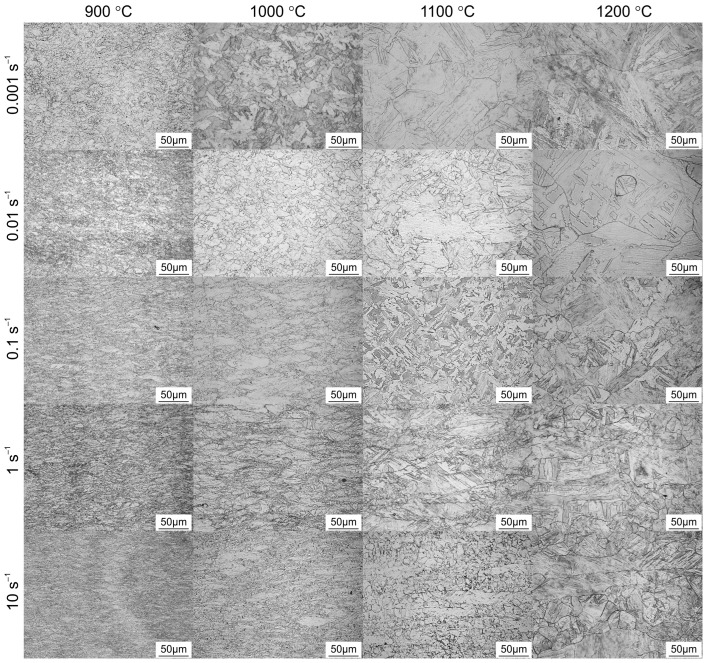
Light microscopy microstructures of hot compression-tested samples of AM 18Ni-300 maraging steel.

The typical optical microscopic microstructures of the additively manufactured 18Ni-300 maraging steel deformed at different deformation conditions are given in Figure 8. For purposes of comparison, all microstructures were captured at the centre of the cross-section of the deformed specimens, and all micrographs were captured at the same magnification. Each microstructure shows the combined effect of temperature and strain rate at the end of deformation.

At the lowest deformation temperature, the microstructure consists mainly of strain-hardened grains (DRV). When the hot deformation temperature increases at a certain strain rate, the dynamically recovered grains are gradually transformed into recrystallized grains as thermally activated processes dominate. When the specimens are deformed in the temperature range of 1100–1200 °C and at a strain rate of 0.001–0.01 s^−1^, the typical deformed grains are replaced by coarsely recrystallized grains (Figure 8).

Similarly, the effect of strain rate is evident at a given hot compression temperature. The material displays flow-softening behaviour at low strain rates and high temperatures because at high temperatures there is enough time to reduce the system’s overall energy [1,20]. whereas in the temperature range of 1100–1200 °C and the strain rate range of 0.1–10 s^−1^, DRX microstructures can be clearly distinguished. It is worth noting that similar behaviour was reported for M350 grade maraging steel [29].

**Figure 9:**
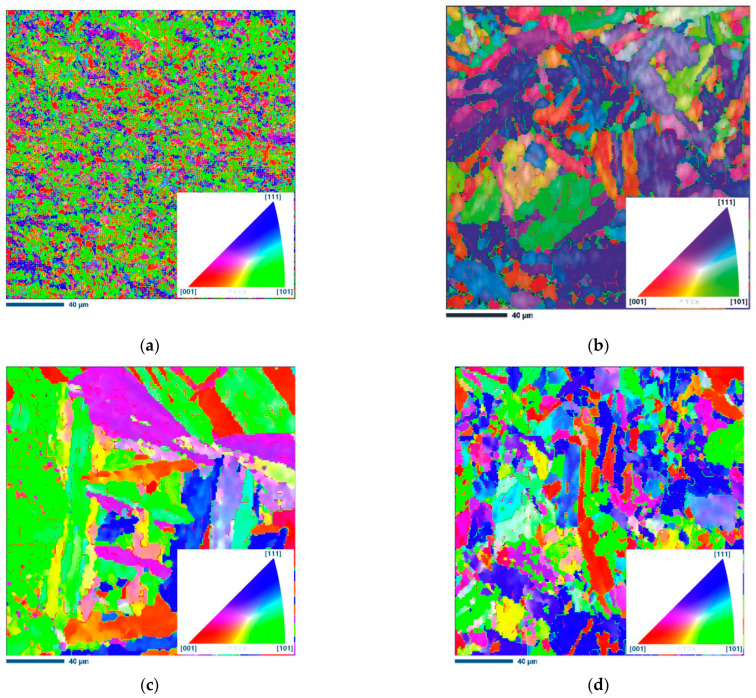
IPF-Z images of 18Ni-300 maraging steel samples (**a**) 900 °C 0.1 s^−1^, (**b**) 1200 °C 0.1 s^−1^, (**c**) 1200 °C 0.001 s^−1^, and (**d**) 1200 °C 10 s^−1^. IPF coloring triangles are shown in the lower right corners of figures, red [001], blue [111] and green [101].

To obtain a more comprehensive characterization of microstructural evolution and changes in grain orientation, EBSD studies were conducted. Figure 9 shows the EBSD maps, where the red lines represent interfaces with a low angle of misorientation (2°–15°), while the green lines represent interfaces with a higher angle of misorientation (>15°).

At a low deformation temperature of 900 °C and a moderate strain rate of 0.1 s^−1^, finer grains with a significant fraction (51.8%) of interfaces with a low angle of misorientation are seen in a microstructure. This relatively high number of LAGBs can be associated with a higher accumulation of dislocations. As shown in Figure 9 and Table 4, when the strain rate is 0.1 s^−1^ and the hot compression temperature is increased to 1200 °C, the percentage of HGABs also increases to 65.1%. This means that the DRX degree is larger at higher deformation temperatures and moderate strain rates than for the specimens deformed at lower temperatures and lower strain rates. The increase in deformation temperature is also accompanied by significant grain growth. As can be seen from Table 4, the grain size increases from 1.8 to 6.5 µm.

At any given deformation temperature, the effect of strain rate is also clearly seen. At the lowest strain rate considered in this study of 0.001 s^−1^ and a hot compression temperature of 1200 °C, the measured average grain size is 8.1 µm. When the strain rate is increased to 10 s^−1^, the grain size decreases to 5.2 µm. Meanwhile, the percentage of HAGBs also increases with increasing strain rate. For the samples with a strain rate of 0.001 s^−1^ at 1200 °C and 10 s^−1^ at 1200 °C, it increased from 65.1% to 76.1%, confirming some occurrence of DRX.

From the inverse pole figures of the specimens deformed at 1200 °C, it can be concluded that the thickness of the martensite plates is also affected by the strain rate. It can be seen that the width of the martensite plates increases with decreasing strain rate. At the highest strain rate of 10 s^−1^, the competition between dynamic recovery and dynamic recrystallization is also evident, so the microstructure consists of relatively thin martensite plates/lamellae with a considerable amount of ultrafine DRX grains (Figure 9d).

**Figure 10:**
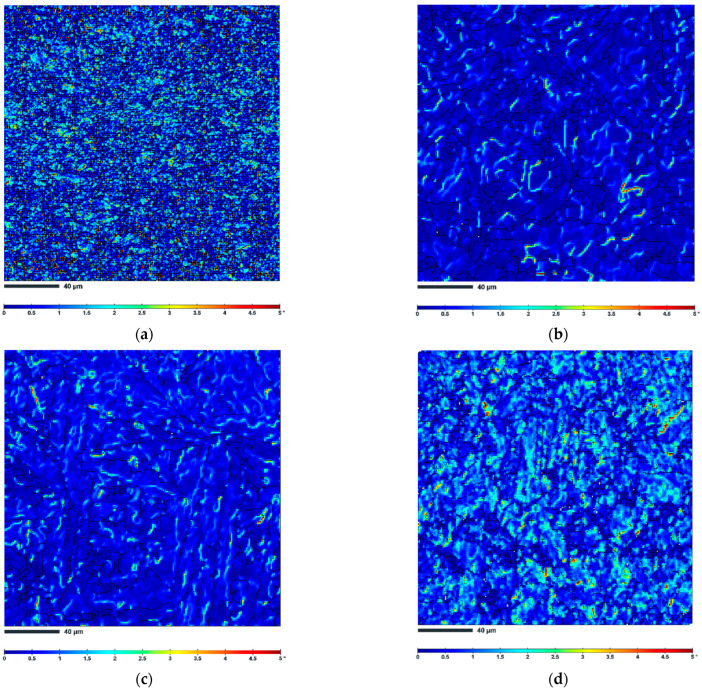
EBSD KAM maps of AM 18Ni-300 maraging steel samples (**a**) 900 °C 0.1 s^−1^, (**b**) 1200 °C 0.1 s^−1^, (**c**) 1200 °C 0.001 s^−1^, (**d**) 1200 °C 10 s^−1^ (black lines correspond to the grain boundaries).

Figure 10 shows the corresponding Kernel Average Misorientation (KAM) maps of selected AM 18Ni-300 maraging steel samples. The high KAM values represent strain accumulation (high stored energy), while the lower KAM values represent (dislocation free) recrystallized grains [30]. As can be deduced from Table 4 and Figure 10a, the highest KAM value of 1.12° shows a specimen compressed at 900 °C and a moderate strain rate of 0.1 s^−1^ with no evidence of dynamic recrystallization. This indicates the presence of a network of accumulated dislocations and confirms the dislocation slip activities [31,32]. It is also worth noting that KAM evolves inhomogeneously in this sample. The highest KAM values are found in the smallest grains and close to the boundaries, which are therefore thought to store the largest amount of dislocations.

The 1200 °C specimen deformed at the same strain rate of 0.1 s^−1^ has a relatively low KAM value of 0.68°, indicating that the higher deformation temperature allows the release of stored deformation energy. It is also worth noting that the effect of strain rate is also clearly visible in the KAM maps. As the strain rate decreases (Figure 10c), the value of KAM also decreases slightly to 0.54°, indicating the lowest dislocation density and the lowest slip activity. On the other hand, the highest value of KAM of 0.98° at a strain rate of 10 s^−1^ indicates the highest dislocation accumulation, which means that the driving force for dynamic recrystallization (DRX) is also highest (the insufficient dislocation density cannot activate dynamic recrystallization process). This is can explain why more dynamically recrystallized grains developed at this strain rate (see Figure 9d).

## 4. Conclusions

Additive manufactured 18Ni-300 maraging steel samples were hot compressed to investigate intrinsic workability, hot working properties and microstructure development. The following conclusions are made based on the above analysis:

- The hot deformation experiments were performed at strains of 0.7 at 900, 1000, 1100, and 1200 °C with initial strain rates of 0.001, 0.01, 0.1, 1, and 10 s^−1^. Typically, the flow stress of the additive manufactured 18Ni-300 maraging steel decreased with increasing temperature and increased with increasing strain rate.
- Dynamic recrystallization is the softening mechanism occurring at high temperatures and at intermediate/high strain rates as confirmed by the EBSD study.
- The constitutive relation corresponding to the peak flow stress is:
  $$\dot{\varepsilon }=7.81\cdot 10^{12}[\sinh{\left(0.0098\sigma \right)]}^{4.13}\exp\left(-\frac{379\cdot 10^{3}}{RT}\right)$$
  in which the activation energy, *Q* = 379 kJ/mol.
- With increasing deformation temperature, the grain size increases, which is accompanied by an increase in the width of the martensite laths.

## Figures and Tables

**Table 1:** Selective laser melting process parameters used for the production of 18Ni-300 steel samples.

Power (W)	Layer Thickness, µm	Laser Speed, mm/s	Hatch Distance, mm
200	30	340	0.12

**Table 2:** Elemental composition of 18Ni-300 maraging steel powder.

Element	Fe	Ni	Co	Mo	Ti	Al	Cr	Cu	C	Mn	Si	P	S
Max %	Bal.	19.00	9.50	5.20	0.80	0.15	0.50	0.50	0.03	0.10	0.10	0.01	0.01
Min %	Bal.	17.00	8.5	4.50	0.60	0.05	–	–					

**Table 3:** Detailed values of material constants and activation energy for the AM 18Ni-300 maraging steel.

Parameter	Value
*α* (MPa^−1^)	0.0098
*n*	4.13
*S*	11.04
*Q* (kJ/mol^−1^)	379
*A*	7.81 × 10^12^

**Table 4:** Main microstructural parameters derived from electron backscatter diffraction.

Sample	f_HAGBs_	f_LAGBs_	Grain Size, µm	Average KAM (°)
900 °C 0.1 s^−1^	48.2	51.8	1.8	1.12
1200 °C 0.1 s^−1^	75.6	24.4	6.5	0.68
1200 °C 0.001 s^−1^	65.1	34.9	8.1	0.54
1200 °C 10 s^−1^	76.1	23.9	5.2	0.98

## Data Availability

All the raw data supporting the conclusion of this paper were provided by the authors.
